# Formulation and Evaluation of SNEDDS Loaded with Original Lipophenol for the Oral Route to Prevent Dry AMD and Stragardt’s Disease

**DOI:** 10.3390/pharmaceutics14051029

**Published:** 2022-05-10

**Authors:** Maxime Vincent, Laurianne Simon, Philippe Brabet, Philippe Legrand, Christophe Dorandeu, Josephine Lai Kee Him, Thierry Durand, Céline Crauste, Sylvie Begu

**Affiliations:** 1 ICGM, Univ Montpellier, CNRS, ENSCM, 34000 Montpellier, France; maxime.vincent@umontpellier.fr (M.V.); laurianne.simon@umontpellier.fr (L.S.); philippe.legrand@umontpellier.fr (P.L.); christophe.dorandeu@umontpellier.fr (C.D.); 2 Institut des Neurosciences de Montpellier, INSERM U1051, 34000 Montpellier, France; 3 Centre de Biochimie Structurale, CNRS UMR 5048, INSERM U1054, 34000 Montpellier, France; josephine.laikeehim@cbs.cnrs.fr; 4 IBMM, Univ Montpellier, CNRS, ENSCM, 34000 Montpellier, France; thierry.durand@umontpellier.fr

**Keywords:** dry age-related macular degeneration (AMD), Stargardt’s disease (STGD1), self-nanoemulsifying drug delivery system (SNEDDS), antioxidant, lipophenol, oral delivery

## Abstract

Dry age-related macular degeneration (Dry AMD) and Stargardt’s disease (STGD1) are common eye diseases, characterized by oxidative and carbonyl stress (COS)-inducing photoreceptor degeneration and vision loss. Previous studies have demonstrated the protective effect of photoreceptors after the intravenous administration of a new lipophenol drug, phloroglucinol-isopropyl-DHA (IP-DHA). In this study, we developed an oral formulation of IP-DHA (BCS Class IV) relying on a self-nanoemulsifying drug delivery system (SNEDDS). SNEDDS, composed of Phosal^®^ 53 MCT, Labrasol^®^, and Transcutol HP^®^ at a ratio of 25/60/15 (*w*/*w*/*w*), led to a homogeneous nanoemulsion (NE) with a mean size of 53.5 ± 4.5 nm. The loading of IP-DHA in SNEDDS (SNEDDS-IP-DHA) was successful, with a percentage of IP-DHA of 99.7% in nanoemulsions. The in vivo study of the therapeutic potency of SNEDDS-IP-DHA after oral administration on mice demonstrated photoreceptor protection after the induction of retinal degeneration with acute light stress (73–80%) or chronic light stress (52–69%). Thus, SNEDDS formulation proved to increase the solubility of IP-DHA, improving its stability in intestinal media and allowing its passage through the intestinal barrier after oral force-fed administration, while maintaining its biological activity. Therefore, SNEDDS-IP-DHA is a promising future preventive treatment for dry AMD and STGD1.

## 1. Introduction

**Figure 1:**
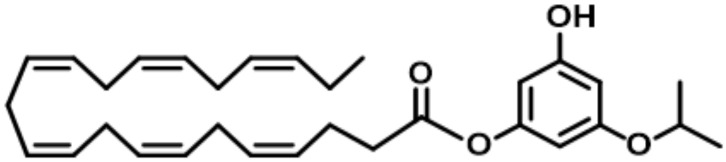
Structure of IP-DHA.

Dry age-related macular degeneration (dry AMD) is a common eye disease that begins at the age of 60 years and will be a major health issue for years to come [1]. As the lifespan of the world’s population is constantly increasing, and with it, the pathologies linked to aging, AMD has affected more than 196 million in 2020 and 288 million in prevision in 2040 [2]. The physiopathology of dry AMD and Stargardt’s genetic disease (STGD1) share a common toxic mechanism involving *trans*-retinal abnormal accumulation in the retinal pigment epithelium (RPE) (reactive aldehyde), and both oxidative and carbonyl stresses (COS), leading to *trans*-retinal metabolization. By undergoing this stress, the *trans*-retinal will be transformed in cytotoxic lipidic compounds, A2E, its photoisomers, and its oxidized metabolites [3]. The accumulation of these compounds can lead to photoreceptor degeneration and vision loss [4]. Currently, there is no treatment for these diseases; only a few molecules with anti COS properties have been proposed as potential candidates to limit the evolution of dry AMD and STDG1 [5]. Based on previous studies [6], a new lipophenol drug, phloroglucinol-isopropyl-DHA (IP-DHA), based on the alkyl-(poly)phenolic omega-3 structure, has been synthetized by our team to prevent these diseases (Figure 1). In vitro experiments on retinal cells demonstrated the absence of the toxicity of IP-DHA, the protective effect of the drug after the induction of oxidative and carbonyl stress, and the importance of the alkyl and the omega-3 chains for efficient cell protection [7]. This drug has also proven its efficacy in vivo on an ABCA4KO (^−/−^) mouse model via an intravenous route (the protection of photoreceptors in the range of 80–85%), and did not affect the retinoid visual cycle [8]. The next step for the development of the treatment is therefore to formulate IP-DHA for oral route administration, to evaluate this formulation in vitro in terms of stability, drug loading, and droplet size, and to prove its in vivo efficacy after inducing retinal degeneration with acute and chronic light stress on ABCA4KO and BALB/c mouse models.

Lipid-based drug delivery systems (LBDDS) can improve the solubility and the oral bioavailability of poorly water-soluble drugs [9,10]. These systems are composed of oils, surfactants, and co-surfactants, and according to the excipients selected and their ratio, LBDDS can be characterized into four types (I, II, IIIA, IIIB, and IV), each presenting with its advantages and disadvantages [11]. Among LBDDS, we can find self-nanoemulsifying drug delivery systems (SNEDDS) that are used to promote the apparent water solubility of hydrophobic drugs [12]. SNEDDS can spontaneously create a homogenous lipid mixture of oil in-water (o/w) nanoemulsions (NE) after being dispersed in water with gentle agitation [13]. The advantage of SNEDDS over other LBDDS, such as solid lipid nanocarriers (SLN), nanostructured lipid nanoparticles (SLN), and liposomes is the lower amount of energy that is required for their preparation, and their high physical stability upon storage and kinetic stability after dispersion in an aqueous phase [13,14,15,16,17,18]. SNEDDS recently proved their efficacy in the prevention of ocular diseases by solubilizing antioxidant compounds such as lutein [19,20] (that share some physical properties with IP-DHA). Diluted in an aqueous phase such as gastric or intestinal fluid and under gentle agitation, SNEDDS allowed for the formation of NE with a smallest droplet size of under 200 nm [21].

In this context, the present study aims to develop and characterize SNEDDS containing IP-DHA (SNEDDS-IP-DHA) for oral administration, in order to enhance the solubility and bioavailability of IP-DHA, and to improve patient compliance [22] for the treatment of dry AMD retinopathy and STGD1. Various SNEDDS formulations were evaluated to select the optimized formulation. Physicochemical tests and phase diagrams were used to select the type and ratio of each excipient to obtain SNEDDS-IP-DHA that yielded to the most suitable NE with a sufficiently high drug-loading rate (30 mg/mL), to allow for in vivo test efficacy at high doses. In this study, SNEDDS were then characterized in vitro (mean size, zeta potential, robustness to dilution, and stability in gastric and intestinal environments). Finally free IP-DHA (solubilized in soybean oil) or SNEDDS-IP-DHA at doses of between 17 and 100 mg/kg were evaluated in terms of the in vivo effectiveness of photoreceptor protection after oral administration, on mice with retinal degeneration induced by acute and chronic light stress.

## 2. Materials and Methods

### 2.1. Materials

IP-DHA was synthetized and purified as previously described [6]. Phosal^®^ 53 MCT was purchased from Lipoïd (Ludwigshafen, Germany). Labrasol^®^ and Transcutol HP^®^ were a gift from Gattefossé (Nanterre, France). Acetonitrile, methanol (MeOH), and sodium chloride (NaCl) were purchased from Honeywell (Charlotte, NC, USA). Pepsin from porcine gastric mucosa, pancreatin from porcine pancreas (4× USP specifications), bile extract porcine, NaOH, and phosphate-buffered saline tablets were purchased from Sigma (Saint Louis, MO, USA). Soybean oil and CaCl_2_ were purchased from Thermo Scientific (Waltham, MA, USA). HCL (37%) was from Fisher Scientific (Pittsburgh, PA, USA). Ultrapure Milli Q water (Millipore, Darmstadt, Germany) was used for SNEDDS dilution. Tropicamide (Mydriaticum 0.5% eye drops, Thea) was used to dilate the pupils, and ketamine (Imalgene 1000, Merial) and xylasine (Rompun 2%, Bayer Healthcare) were used to anesthetize the mice.

### 2.2. Characterization of IP-DHA Properties

#### 2.2.1. Thermal Analysis

IP-DHA was analyzed using thermogravimetric analysis (TGA) and differential scanning calorimetry (DSC), using a Mettler TGA 2 apparatus and Mettler DSC 3+ (Mettler Toledo, USA), respectively. A total of 3 mg IP-DHA was placed in an aluminum plate. A thermogram was recorded from 30 to 400 °C for TGA analysis, and curves of DSC were recorded from 30 to 300 °C for DSC analysis, with a heating rate of 10 °C ·min^−1^. A nitrogen purge at 50 mL ·min^−1^ was maintained over the samples.

#### 2.2.2. X-ray Diffraction Analysis (XRD)

IP-DHA were analyzed via XRD using a AX2 D2 diffractometer (Brucker, USA) with Cu Kα radiation (30 kV, 10 mA) and θ-θ geometry, using a lynxeye detector with an angular range of 5 to 42° 2θ. A total of 2 mg IP-DHA was lightly pressed on a silicon wafer to obtain a flat surface. Data was interpreted using DIFFRAC.EVA software.

#### 2.2.3. µHPLC-MS/MS Analysis

Quantitative analysis was performed on a µHPLC (Sciex, ExionLC AD), and a C18 column (phenomenex C18 luna polar, 30 × 2.1 mm ID, 100 A) was used as the stationary phase. The mobile phase consisted of 0.1% formic acid in water (phase A) and 0.1% formic acid in acetonitrile (phase B), with a gradient mode from 30/70% (*v*/*v*) at 0 min to 0/100 at 1.90 min and to 30/70 at 3 min, respectively (a flow rate of 0.7 mL ·min^−1^) for 10 min. Samples were analyzed on a Sciex 5500 triple-quadrupole MS with selective reaction monitoring, positive ionization mode, a capillary temperature of 300 °C, and a capillary voltage of 5.5 Kv.

#### 2.2.4. Caco-2 Permeability

Caco-2 cells are human intestinal epithelial cells that are derived from a colorectal adenocarcinoma. Cells were seeded at 1 × 10^5^ cells/cm^2^ in 96-well Multiscreen^TM^ plates (Millipore). This assay was performed in either the apical-to-basolateral (A–B) or B–A direction. IP-DHA was prepared at 10 μM in HBSS-MES (pH 6.5) or HBSS-HEPES (pH 7.4), with a final DMSO concentration of 1%. The working solution was then centrifuged, and the supernatant was added to the donor side. The assay plate was incubated at 37 °C with gentle shaking for 60 min or 40 min for the A–B or B–A assays, respectively. Samples were aliquoted from the donor side at time zero and from the end point, and from the receiver side at the end point. Samples were analyzed via HPLC-MS/MS, as described in Section 2.2.3.

The apparent permeability coefficient (*P_app_*) in cm/s of IP-DHA was calculated as follows:

$$P_{app}=\frac{V_{R}\times C_{R\left(end\right)}}{\Delta t}\times \frac{1}{A\times \left(C_{D\left(mind\right)}-C_{R\left(mind\right)}\right)}$$

where *V_R_* is the volume of the receiver chamber. *C_R_*_(*end*)_ is the concentration of IP-DHA in the receiver chamber at the end time point, Δ*t* is the incubation time, *A* is the surface area of the cell monolayer, and *C_D_*_(*mid*)_ is the calculated mid-point concentration of the test compound on the donor side, which is the mean value of the donor concentration at time 0 min and the donor concentration at the end time point. *C_R_*_(*mid*)_ is the mid-point concentration of the test compound in the receiver side, which is one half of the receiver concentration at the end time point.

#### 2.2.5. Stability on Human Microsomes

The metabolic stability of IP-DHA on human microsomes (microsomes S9, cryopreserved hepatocytes, recombinant CYP, and recombinant UGT) has been evaluated and expressed as percentage of IP-DHA remaining. IP-DHA was pre-incubated with pooled liver microsomes in phosphate buffer (pH 7.4) at 10 mM for 5 min in a 37 °C shaking water bath (IKA^®^ RH basic KT/C). The reaction was initiated using the NADPH-generating system and incubated for 0, 15, 30, 45, and 60 min. The reaction was stopped by transferring the incubation mixture to acetonitrile/methanol (1:3 *v*/*v* ratio). Samples were then mixed and centrifuged at 3000 rpm (Sigma centrifuge 2K15) for 20 min to precipitate the protein, and supernatants were collected. IP-DHA concentration was determined using µHPLC-MS/MS analysis, as described in Section 2.2.3. The half-life (T_1/2_) was estimated from the slope of the initial linear range of the logarithmic curve of the compound remaining (%) vs. time, assuming first-order kinetics.

#### 2.2.6. Solubility of IP-DHA

Solubility was evaluated in PBS (pH 7.4), SGF, and ISF (Table 1) without enzymes (pepsin and pancreatin). An excess of IP-DHA was added for each medium type, followed by mixing using a vortex mixer (Vortex Génie 2) and equilibration at room temperature. Next, the mixtures were centrifuged at 3000 rpm (Sigma centrifuge 2K15) for 15 min. The supernatant from each sample was diluted with methanol. The concentration of IP-DHA in each sample was determined using the µHPLC/MS-MS analysis, as described in Section 2.2.3.

### 2.3. Development and Characterization of the SNEDDS Formulation

#### 2.3.1. Pseudo Ternary Phase Diagram: Experimental Design and Formulation of SNEDDS

The suitable excipients selected from the solubility study (Phosal^®^ 53 MCT, Labrasol^®^, and Transcutol HP^®^) were studied to design the optimal SNEDDS formulation in terms of droplet size and physical stability. Different ratios of Phosal^®^ 53 MCT used as an oil base, Labrasol^®^ used as a surfactant, and Transcutol HP^®^ used as a co-surfactant (% *w*/*w*/*w*), were evaluated in a pseudo-ternary phase diagram. A nanoemulsion was obtained after 1:1000 (*v*/*v*) dilution of the lipid mixture composed of Phosal^®^ 53 MCT, Labrasol^®^, and Transcutol HP^®^ in MilliQ water. The formulation was vortexed (Vortex Génie 2) until a homogeneous liquid was obtained. The concentration of the oil phase varied over a range of 5–35 (% *w*/*w*), the surfactant varied from 50 to 85 (% *w*/*w*), and the co-surfactant varied from 5 to 20 (% *w*/*w*).

#### 2.3.2. Preparation and Characterization of SNEDDS-IP-DHA

Different amounts of IP-DHA (from 10 to 40 mg/mL) were first totally solubilized in Phosal^®^ 53 MCT, and then Labrasol^®^ and Transcutol HP^®^ were added at a ratio of 25/60/15 (% *w*/*w*/*w*) in a final volume of 100 µL. The mixture was vortexed (Vortex Génie 2) until a homogeneous liquid phase was obtained. The solubility of IP-DHA in each SNEDDS was determined via HPLC analysis, as described above in Section 2.2.3. SNEDDS formulations that allowed for the highest solubility of IP-DHA were selected for further studies of stability. SNEDDS were diluted in water (1:1000 *v*/*v*) under gentle agitation to obtain NE. The mean size and zeta potential of NE were analyzed using Zetasizer NanoZS, as described in Section 2.3.3.

#### 2.3.3. Determination of the Mean Size and Zeta Potential of the Nanoemulsion

The mean size and zeta potential of the formulations obtained after 1:1000 (*v*/*v*) dilution in MilliQ water obtained from empty SNEDDS and SNEDDS-IP-DHA were investigated using photon correlation spectroscopy (PCS) with a Zetasizer NanoZS apparatus (Malvern Instruments Ltd., Worcestershire, UK) equipped with a He-Ne laser (632.8 nm) at a temperature of 20 °C and a scattering angle of 173° for detection. For the determination of particle size and size distribution, each sample of NE was placed into a cuvette. The particle size was expressed as an average diameter, whereas the particle size distribution was described with the polydispersity index (PDI). For the determination of the zeta potential, each NE was transferred into DT51070 folded capillary cells (Malvern, Worcestershire, UK). All experiments were performed in triplicate.

#### 2.3.4. Morphological Studies

Morphological analysis was performed using cryo-TEM. A total of 3 µL of SNEDDS-IP-DHA at 30 mg/mL in water or in PBS (pH 7.4) were applied to glow-discharged Lacey grids (Delta Microscopies), blotted for 1 s, and then flash-frozen in liquid ethane using a CP3 cryo-plunge (AMETEK SAS—Division GATAN). Before freezing, the humidity rate was stabilized at approximately 95%. Cryo-TEM observation was conducted on a JEOL 2200FS FEG microscope (JEOL Europe SAS) operating at 200 kV under low-dose conditions (total dose of 20 electrons/Å^2^) in zero-energy-loss mode with a slit width of 20 eV. Images were taken at a nominal magnification of 50,000× corresponding to a calibrated magnification of 45,591×, with the defocus ranging from 1.5 to 2 μm with a slow-scan 4K × 4K CCD camera (AMETEK SAS–Division GATAN).

#### 2.3.5. Drug Loading and Percentage of IP-DHA in Nanoemulsions

The drug quantification in NE was conducted via high performance liquid chromatography (HPLC) on an LC6-2012HT model apparatus (Shimadzu, Kyoto, Japan) with a Prontosil C8 (120-5-C8 SH, 5 µm, 250 × 4.0 mm) column coupled with a UV-visible detector (Shimadzu, Kyoto, Japan). The flow rate was set to 1 mL/min, and the detection was measured at 206 nm. The mobile phase was a mixture of water and acetonitrile used in isocratic mode with a 90/10 acetonitrile/water ratio. The calibration curve line was linear, from 1 to 50 µg/mL, with a correlation coefficient of r² = 0.9996. All experiments were performed in triplicate.

The drug loading was calculated using the following equation:

$$\mathrm{Drug}\;\mathrm{Loading}=\frac{\mathrm{Amount}\;\mathrm{of}\;\mathrm{IP}-\mathrm{DHA}\;\mathrm{added}}{\mathrm{Total}\;\mathrm{amount}\;\mathrm{of}\;\mathrm{IP}-\mathrm{DHA}+\mathrm{excipients}}$$

The percentage of IP-DHA in the nanoemulsions was determined according to the following equation:

$$\%\;\mathrm{of}\;\mathrm{IP}-\mathrm{DHA}\;\mathrm{in}\;\mathrm{nanoemulsions}\;=\frac{\mathrm{Amount}\;\mathrm{of}\;\mathrm{IP}-\mathrm{DHA}\;\mathrm{added}}{\mathrm{Amount}\;\mathrm{of}\;\mathrm{IP}-\mathrm{DHA}\;\mathrm{dosed}}\times 100$$

#### 2.3.6. Robustness to Dilution

SNEDDS and SNEDDS-IP-DHA at 30 mg/mL were diluted in MilliQ water at 1:1000, 1:750, 1:500, 1:250, 1:100, and 1:10 (*v*/*v*). The samples were analyzed using DLS after magnetic agitation (150 rpm) for 5 min.

#### 2.3.7. Influence of the SNEDDS Formulation on IP-DHA Stability in Gastric- and Intestinal-Simulated Fluids

The chemical stability of free IP-DHA and IP-DHA loaded in SNEDDS at 30 mg/mL was evaluated in a gastric and an intestinal environment (Table 1). A total of 150 µL of free IP-DHA (solubilized in MeOH) or SNEDDS-IP-DHA loaded at 30 mg/mL were placed in 20 mL of freshly prepared SGF or ISF under magnetic stirring at 150 rpm in a water bath at 37 °C. Samples were collected at 0, 30, 60, and 90 min for the stability analysis in SGF, or 0, 20, 40, 60, 80, 100, and 120 min for the stability analysis in ISF. A total of 500 µL of the medium was recovered and diluted with 500 µL of MeOH to stop the enzymatic degradation reaction. The samples were centrifuged (Sigma centrifuge 2K15) for 10 min at 15,000 rpm. The supernatant was removed and IP-DHA was quantified as described in Section 2.3.5.

### 2.4. In Vivo Evaluation of Photoreceptor Protection of IP-DHA and SNEDDS-IP-DHA on Mice after Oral Administration

#### 2.4.1. Animals

A total of 85 ABCA4^−/−^ albino mice were purchased from Taconic Biosciences Inc. on a mixed 129/S5 × C57BL/6J-Tyrc-Brd background. The mice were bred and genotyped for the Abca4 null mutation, and were homozygous for Rpe65-Leu450. The rd8 mutation was absent in this ABCA4KO line. A total of 36 BALB/c that were 8 weeks of age were purchased from Charles River. Mice were subjected to standard light cycles of 12 h of light (90 Lux) and 12 h of darkness, at a room temperature that was close to 22 °C, and fed ad libitum with a standard rodent diet. The mice were housed in facilities that were accredited by the French Ministry of Agriculture and Forestry (n°C34-17236—19 December 2014). Experimental procedures were conducted in accordance with the European directive, 2010/63/EU, and the French Ministry of Education and Research (APAFIS n°15117-2018051712092842 v3), concerning the care and use of the animals.

#### 2.4.2. Protocol Administration

Mice between 8 and 16 weeks of age (both sexes), were weighed to adjust the quantity of administered compound for a dosage at 100, 50, or 17 mg/kg. Mice were orally fed with a 22-gauge needle containing SNEDDS or soybean oil as a control vehicle, IP-DHA solubilized in soybean oil (20 mg/mL), and SNEDDS-IP-DHA (30 mg/mL).

#### 2.4.3. Acute Light-Induced Retinal Degeneration on Abca4KO Mice

After the mice were dark-adapted (DA) for 24 h, their pupils were dilated with 0.5% tropicamide. The mice were force-fed in the dark 1 h before light exposure. Mice were exposed to constant light (two cool white fluorescent lamps, OSRAM of 26 watts, 1800 lm, a maximum photopic efficiency of ~470 nm, light intensity averaged to 24 mW/cm^2^, corresponding to ≈20.000 Lux) for 1.50 h in a white plastic bucket with a fan. Mice were kept in the dark for 5 days before we performed the final retinal evaluations. Throughout the experiment, the ambient temperature was 22 °C, which was identical to that of the breeding box, to avoid an increase in body temperature, which could further damage the retina.

#### 2.4.4. Chronic Light-Induced Retinal Degeneration on BALB/c Mice

Mice were maintained under a blue light (165 w, full-spectra LED light dimmable, C-Disount) ≈1000 Lux for 7 days with a 12/12 h light/dark cycle in a plastic cage. Mice were fed once a day 1 h before light exposure. Mice were fed twice a day 1 h before light exposure, and 6 h after the first administration. Mice who were fed three times/day were fed 1 h before light exposure, and 4 and 8 h after the first administration. At the end of the 7 days, the mice were placed in the dark for 5 days before the analysis of their retinas. Throughout the experiment, the ambient temperature was 22 °C, identical to that of the breeding box, to avoid an increase in body temperature, which could further damage the retina.

#### 2.4.5. Histological Analysis

All animals were killed via cervical dislocation. The eyes were rapidly enucleated and fixed in 4% paraformaldehyde for 24 h at 4 °C. Eyecups were embedded in paraffin and cut into 5 μm sagittal sections. For hematoxylin/eosin/saffron (HES) staining, the sections were deparaffined, labeled with HES using standard protocols, rinsed, and mounted in Moeviol. The thickness of the inner nuclear layer (INL) and the outer nuclear layer (ONL) was measured and expressed in arbitrary units using ImageJ software. Thickness measurements were made manually on the central part of the retina (near the optic nerve). A total of 20 measurements were made per eye (10 for the ONL measurement and 10 for the INL measurement), and these values were averaged to calculate the ratio of INL/ONL thickness.

#### 2.4.6. Electroretinography

All electrophysiological examinations were conducted using the Visiosystem (SIEM, France). Animals were prepared and anesthetized, and electroretinogram recording (binocular full-field ERG) was performed with cotton electrodes, as previously described [23].

#### 2.4.7. Statistics

Statistical analyses were performed using Graph Pad Prism 5.0 software. One-way ANOVA was used to compare more than two samples. Bonferroni’s multicomparison adjustment was used as the post hoc test to calculate the significance levels. A *p*-value of <0.05 was considered significant.

## 3. Results and Discussion

Dry age-related macular degeneration (AMD) and Stargardt disease (STDG1) undergo a known toxic mechanism caused by carbonyl and oxidative stresses (COS). This is responsible for the accumulation in the retinal pigment epithelium (RPE) of A2E, which is a main toxic pyridinium bis-retinoid lipofuscin component. This accumulation leads to the death of photoreceptors, outer retina cells, and retinal pigment epithelium. The molecular and cellular mechanisms of these diseases have previously been described [24,25]. Previous studies have shown that carbonyl stress in retinal cells could be reduced through an original alkyl-phloroglucinol-DHA conjugate (lipophenol) [6]. This new molecule was developed starting from the phloroglucinol base, which is known for its antioxidant properties (an active compound of Spasfon^®^), and potential therapeutics for other neurodegenerative diseases [26]. On this basis, an isopropyl function was grafted to improve the nucleophilicity nature of the aromatic ring that should be involved in the detoxification of the toxic aldehyde concentration. Finally, to promote the targeting of the molecule to the outer retina, we also grafted docosahexaenoic acid (DHA, C22:4 n-3), a polyunsaturated fatty acid that is localized in the photoreceptor disc membrane and that is known to have several benefits for retina protection and function [27]. As the mechanism of action for IP-DHA has been previously discussed [8,28], this present work focused on the impact of the IP-DHA features on its resulting activity based on photoreceptor protection. Thus, the study then led to the development of the formulation best suited for oral delivery. This was followed by the evaluation of the therapeutic efficacy of the loaded formulation, which was then investigated in vivo on mice using a light-induced photoreceptor degeneration model.

### 3.1. Characterization of IP-DHA Properties (Pre-Formulation Studies)

To select the best formulation for the oral delivery of IP-DHA, we first conducted a broad investigation on its features, such as its solubility in various media, and its permeability, using in vitro models. The solubility of IP-DHA was evaluated in phosphate-buffered saline (PBS), and in gastric- and intestinal-simulated fluids (SGF and SIF, respectively). Overall, IP-DHA was found to be poorly soluble in all media (Table 2); in PBS, at 11.54 ± 1.53 µg/mL, in ISF at 10.19 ± 0.12 µg/mL, and even more poorly in SGF, at 7.39 ± 0.26 µg/mL. These results confirmed the poor solubility of IP-DHA and the need for a formulation to increase its solubility. Regarding the permeability test, as IP-DHA presented a high log P value (>5) (Table 2), it was safe to assume that this should lead to a low permeability vs. physiological membrane. To confirm this hypothesis, permeability studies on human intestinal cells, Caco-2, were conducted (Table 2). The diffusion of IP-DHA from apical to basolateral was found at 1.0 ± 0.14 10^−6^ cm/s, and from basolateral to apical, at 0.7 ± 0.05 10^−6^ cm/s. This illustrated the low degree of diffusion of the drug, and it is considered to be a low-permeability drug [29]. As IP-DHA is poorly soluble and poorly permeable, it can be classified as a BCS Class IV drug [30]. Moreover, in vitro metabolic stability tests analyzed on hepatic microsomes (Table 2) highlighted an important first-pass hepatic metabolization of IP-DHA (Figure S3).

To complete the solubility and permeability study, the physical properties of IP-DHA were analyzed via X-ray diffraction (XRD), thermogravimetric analysis (TGA), and differential scanning calorimetry (DSC). XRD analysis (Figure S1) revealed that no sharp diffraction peak indicated the amorphous nature of IP-DHA. TGA analysis (Figure S2) showed a decrease in the weight of IP-DHA from 240 °C, corresponding to a decomposition without any loss at 100 °C. This result confirmed the anhydrous nature of IP-DHA. Then, the DSC analysis (Figure S2) illustrated a large broad endothermic peak at 256 °C, potentially due to decomposition (melting point). Overall, the amorphous state of IP-DHA was considered to be stable, as no recrystallization was observed during DSC. Based on this full characterization of the IP-DHA properties, the formulation of the drug was designed.

### 3.2. Development of the SNEDDS Formulation

#### 3.2.1. Choice of SNEDDS and Composition

Poorly water-soluble drugs such as IP-DHA must have their apparent solubility and permeability improved in order to increase oral bioavailability and thus, therapeutic efficacy. This can be achieved by designing the best-suited formulation. Looking at the literature, LBDDS formulations were proven to enhance the pharmacokinetics of the BCS class IV drug, through a variety of effects on their solubility and distribution. Among the different LBDDS, we selected SNEDDS as they can improve the oral bioavailability of BCS class IV drugs, increase drug solubility, and reduce the hepatic first-pass metabolism bypass via lymphatic absorption [31,32,33] using smart excipients [34]. This last feature is of interest, as we previously highlighted an important aspect of first-pass hepatic metabolism. SNEDDS is an isotropic mixture that is composed of oil, surfactant, and co-surfactant, which form an oil-in-water NE in contact with aqueous media such as gastric or intestinal fluid [35]. The oil phase improves IP-DHA solubility, and then lipophilic and hydrophilic surfactants reduce the size of the NE droplets by decreasing the surface tension with the gastrointestinal fluid [36].

Among the excipients of SNEDDS selected to improve IP-DHA bioavailability, we chose Phosal^®^ 53 MCT for the oil phase. It is composed of phosphatidylcholine (53%) and medium-chain triglycerides (MCT), and the solubility of IP-DHA in it was found to be high, at 25.47 ± 3.27 mg/mL. Moreover, MCT is known to be absorbed directly by epithelial cells in the gastrointestinal tract, improving membrane penetration and permeability. Our hypothesis of mechanism also relies on the presence of lipids in the duodenum that will lead to the secretion of cholesterol and bile salts. This induces the formation of colloid micelles that are able to solubilize lipophilic compounds such as IP-DHA [37]. Then, lipid digestion stimulates the production of chylomicrons. Lipidic compounds such as IP-DHA in association with the triglyceride core of chylomicrons are secreted into the lymph by exocytosis [38]. It was shown that compounds with high logP (>5) could preferentially pass to the lymphatic pathway via entrapment in chylomicrons [39]. Labrasol^®^ solubilized IP-DHA at 46.00 ± 2.15 mg/mL, and thus it was selected as a non-ionic surfactant. It is composed of a small fraction of mono-, di-, and triglycerides, and mainly PEG-8 mono- and diesters of caprylic (C8) and capric (C10) acids with a high HLB value (12). The use of non-ionic surfactant was preferable, as it was proven to be less toxic and less of an irritant than the ionic surfactant. Labrasol^®^ reduced the surface tension of oil droplets formed at the oil/water interface of the NE, and it is also well known to increase the fluidity of membrane [40] and paracellular transport [41]. Finally, Transcutol HP^®^ (diethylene glycol monoethyl ether) (HLB value ≈ 4) was used as an amphiphilic co-surfactant to stabilize NE droplets, and moreover, it increases the solubility of IP-DHA at 45.02 ± 2.16 mg/mL and will increase membrane fluidity [42]. Furthermore, Transcutol HP^®^ could inhibit the P-gP efflux pump [43]. All of these mechanisms should induce an increase in the bioavailability of IP-DHA.

#### 3.2.2. Effect of the SNEDDS Composition on Droplet Size for Ratio Selection

**Figure 2:**
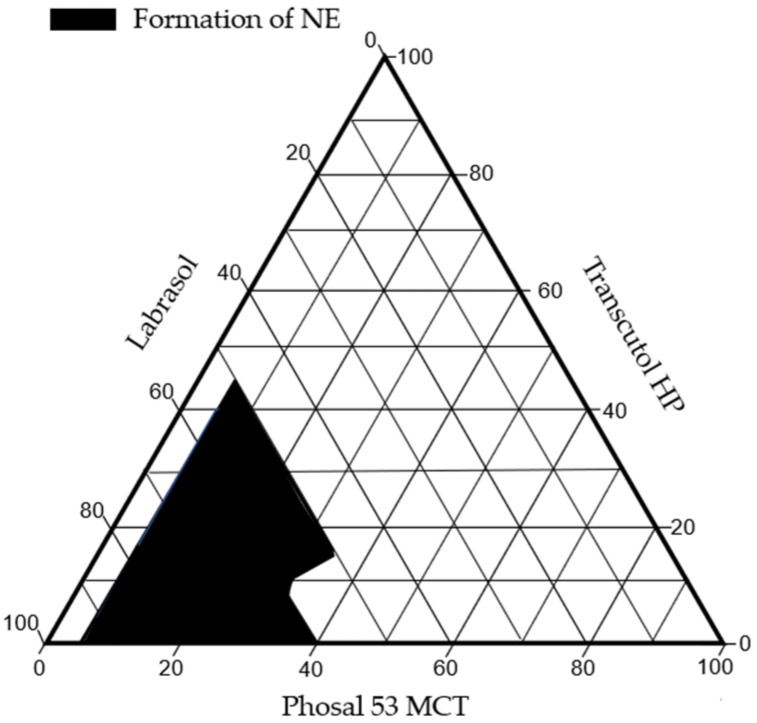
Pseudo-ternary phase diagram.

Generally speaking, droplet size is an important factor for the improvement of drug dissolution [44]. Indeed, small droplet sizes will induce a larger exchange surface for IP-DHA, and then promote higher drug absorption [45]. Thus, we aim to obtain the smallest and most stable NE. For this, a pseudo-ternary phase diagram evaluation was performed to determine the ratio of oil/surfactant/co-surfactant (Figure 2). This was optimized by observing the effect of various concentrations of these excipients on the mean size, with DLS measurements (Table 3).

Therefore, the ratio of oil phase/surfactant/co-surfactant of 25/60/15 (*w*/*w*/*w*) was able to form the smallest NE, and this has been selected as the optimized SNEDDS formulation for this study.

#### 3.2.3. Determination of the Maximum Drug Loading of IP-DHA in SNEDDS

**Figure 3:**
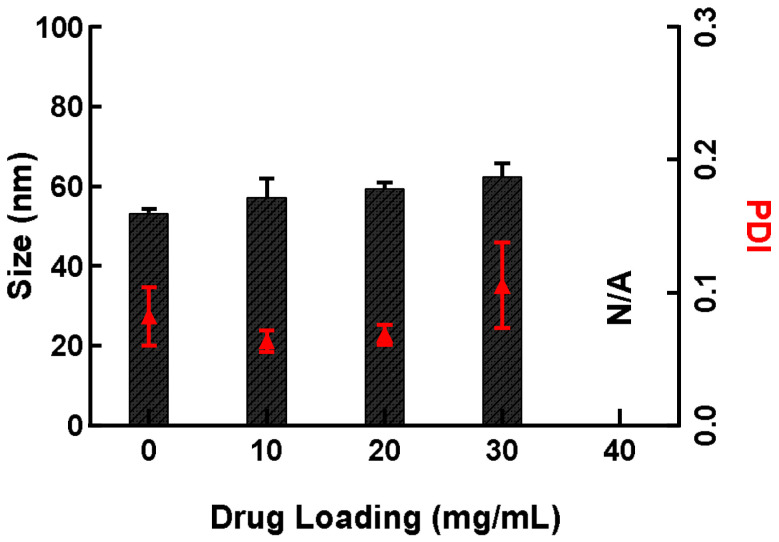
The influence of IP-DHA drug loading on the mean size of NE after the dilution of SNEDDS-IP-DHA in MilliQ water (dilution 1:1000). The columns represent the mean size (nm) and the red triangles the PDI. (*n* = 3).

To fully validate this choice of ratio, the SNEDDS were then loaded with our drug of interest, IP-DHA. Previous studies have demonstrated a maximum efficacy of IP-DHA associated with BSA at 30 mg/kg in mice, using the IV route [8]. Based on these results, we aimed at formulating IP-DHA loaded in SNEDDS to experiment with oral administration using higher IP-DHA doses (around 100 mg/kg). The objective was to evaluate the maximum amount of IP-DHA that could be loaded with SNEDDS forming homogenous NE, in order to realize dosage responses using the oral administration process in animals. Therefore, we performed a study to determine the maximum degree of drug loading of IP-DHA in the SNEDDS while maintaining the droplet size and PDI at a low rate. Different amounts of IP-DHA were loaded (10, 20, 30, and 40 mg/mL) in the SNEDDS formulation, with a previously selected ratio of 25/60/15. The droplet size of NE was evaluated through DLS measurement after the dilution of SNEDDS-IP-DHA in water (Figure 3). The results showed a similar mean size: 57.22 ± 4.72 nm, 59.46 ± 1.56 nm, and 62.40 ± 3.37 nm for SNEDDS at 10, 20, and 30 mg/mL, respectively. Over this concentration (40 mg/mL), the NE were heterogeneous (PDI > 0.3).

Therefore, SNEDDS-IP-DHA at 30 mg/mL has been selected to pursue the experiments.

#### 3.2.4. Drug Loading and Percentage of IP-DHA in Nanoemulsions

The drug loading (DL) and the percentage of IP-DHA in nanoemulsions were calculated from the equation described in Section 2.3.5. Even if the percentage of IP-DHA inside the NE compared to the amount of IP-DHA present in solution was close to 100% (99.08 ± 0.85%), we observed a low amount of IP-DHA that was loaded (2.93 ± 0.12%) relative to the total lipid excipients of the SNEDDS formulation. The drug loading was in accordance with the desired concentration of IP-DHA in SNEDDS (30 mg/mL) for preclinical efficiency evaluation.

### 3.3. NE Characterization

#### 3.3.1. Size, Zeta Potential Analysis, and Morphological Studies

Overall, the SNEDDS formulation, with a selected ratio of oil phase/surfactant/co-surfactant of 25/60/15 was able to perform NE loading with IP-DHA at 99% while maintaining a small droplet size and a low PDI. Indeed, the formulation characterization of empty NE and NE-IP-DHA (Table 4) summarized their size and zeta potential. IP-DHA led to an increase of the mean size, from 53.53 ± 4.50 to 62.40 ± 3.37 nm, with the PDI value remaining stable (<0.3). These values confirmed the presence of drug loading in NE droplets. Regarding the zeta potential, NE and NE-IP-DHA had a zeta potential of −8.04 ± 1.73 and −22.03 ± 3.29 mV.

**Figure 4:**
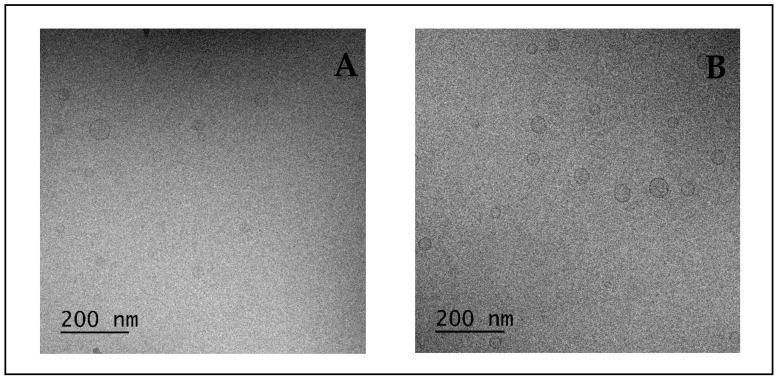
NE from SNEDDS 30 mg/mL diluted at 1:100 in MilliQ water (**A**) or PBS (pH 7.4) (**B**) from cryo-TEM images (50,000×).

The zeta potential informs us of the potential stability of NE. NE with a zeta potential value of ±30 mV was considered to be stable when diluted in aqueous media, due to electrostatic repulsion between the NE, which limits coalescence [46]. We supposed that the negative charge of NE was probably due to the ionization of free fatty acids of labrasol [47] and the increase of this negative charge of NE-IP-DHA compared to empty NE was probably due to the ionization of the phenolic OH- group from IP-DHA [10]. This could be an indicator of the localization of IP-DHA at the O/W interface, suggesting that IP-DHA was not fully encapsulated inside NE, but adsorbed at the interface or surface. The fact that we exceeded the ±30 mV threshold could have an impact on the stability of NE; however, no precipitation was visually assessed during the in vitro experiments, and the droplet size and PDI remained stable. To conclude, regarding the evaluation of the formulation, the morphological shape of NE was determined via cryo-TEM imaging (Figure 4). Cryo-TEM results further confirmed the spherical shape, the vesicular morphology, and the unilamellar structure of NE obtained after the dilution of SNEDDS in MilliQ water or PBS (pH 7.4). NE observed after its dilution in MilliQ water (Figure 4A) and in PBS (pH 7.4) (Figure 4B) were similar in terms of droplet size. The ionic strength carried by the PBS had no significant impact on the droplet size of the NE. The preparation method of the samples for cryo-TEM (dehydration) should explain the variation of droplet size compared to DLS analysis.

#### 3.3.2. Robustness to Dilution

**Figure 5:**
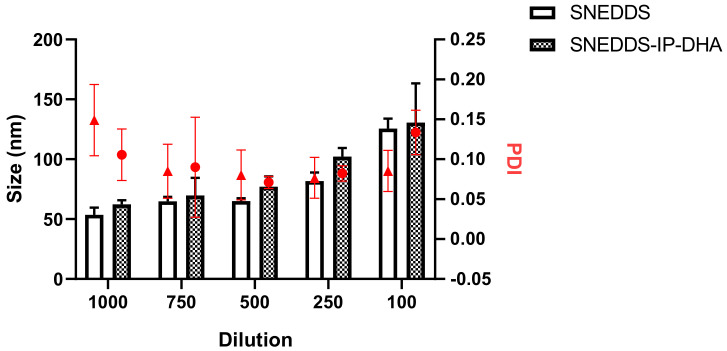
Mean size and PDI of empty NE and NE-IP-DHA as a function of water dilution of SNEDDS and SNEDDS-IP-DHA at 30 mg/Ml. The columns represent the mean size (nm) and the red circles or triangles the PDI. (*n* = 3).

To finish the full characterization of the SNEDDS, we first conducted a stability study for dilution in water, looking at the size of the droplets, and again in the presence of gastric and intestinal fluids. Indeed, the volume of fluids in the gastrointestinal tract varies during the day [48] as it increases after a meal (with the secretion of digestive enzymes). Thus, it is a matter of particular importance to determine the capacity of the SNEDDS to form a homogenous NE, depending on the volume of aqueous medium. For this, we diluted the SNEDDS in water by a factor ranging from 100 to 1000. We observed that mean size of the NE varied as a function of the volume of water used for SNEDDS dilution. Between 1:1000 and 1:500, dilution induced a low impact in terms of droplet size variation, as we observed 62.40 ± 3.37 nm at the 1000th dilution, and 77.03 ± 8.79 nm at the 500th dilution with SNEDDS-IP-DHA. At a lower dilution, we observed an increase of droplet size with 102.24 ± 7.22 nm and 130.65 ± 32.79 nm, at a dilution of 1:250 and 1:100, respectively (Figure 5, blank column). SNEDDS-IP-DHA (Figure 5, gray column) formed a slightly larger NE than those obtained with empty SNEDDS due to the presence of IP-DHA. The droplet size of NE from SNEDDS increased with decreasing dilution volume in the same way as SNEDDS-IP-DHA. We did not observe any precipitation or phase separation during the experiment.

We concluded that the volume of water has an impact on the mean size of the NE formed, but they were homogeneous in terms of polydispersity (PDI < 0.3). The fact that the droplet size of the NE increases with the decreasing of the volume media may have an impact on the drug absorption. It will be preferable to use dilution conditions of 1:1000 to optimize the absorption of IP-DHA.

#### 3.3.3. Influence of SNEDDS on IP-DHA Stability in Gastric- and Intestinal-Simulated Fluids

**Figure 6:**
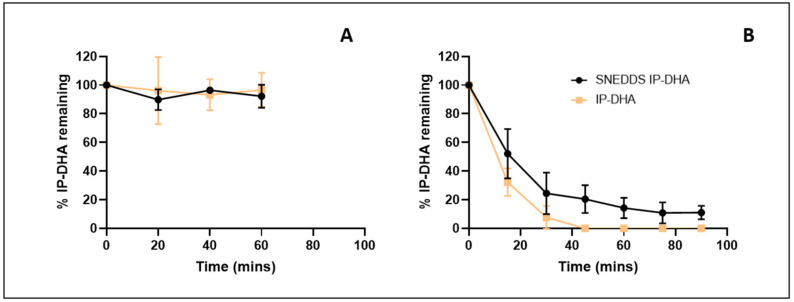
Stability of IP-DHA in SGF (**A**) or ISF (**B**) after dilution of free IP-DHA or SNEDDS-IP-DHA at 30 mg/mL (*n* = 3).

The stability of IP-DHA, SNEDDS, and SNEDDS-IP-DHA was studied in SGF and SIF media to evaluate the impact of pH and enzymes on the potential degradation of IP-DHA over time. In the gastric medium, with or without formulation, IP-DHA remained stable and was not degraded by a low pH or by pepsin in the medium (Figure 6A). Without formulation, the solubility of IP-DHA in the gastric medium was limited (7.39 ± 0.26 µg/mL). This solubility was increased when using the SNEDDS formulation (>225 µg/mL). In the intestinal environment, with and without formulation, a degradation of IP-DHA was observed (Figure 6B). This degradation was probably due to the presence of pancreatin in the environment. We presumed that pancreatin could cleave the ester function of IP-DHA. However, this degradation is limited when using SNEDDS (T_1/2 SNEDDS-IP-DHA_ = 17 min vs. T_1/2 IP-DHA_ = 11 min). Similar to SGF, the solubility of IP-DHA in the ISF (>225 µg/mL) was increased when using the SNEDDS formulation. These results were not surprising; it has been reported that SNEDDS may protect drugs from enzymatic degradation in the gastrointestinal tract [21]. Moreover, another study showed that nonionic surfactants provide protection against lipase destabilization in emulsions [49], which could explain the limitation of the degradation of IP-DHA with SNEDDS.

SNEDDS resultantly enhanced the solubility of IP-DHA in SGF and ISF. The low pH and the presence of pepsin from SGF had no impact on the amount of IP-DHA recovered. The degradation of IP-DHA in ISF caused by intestinal enzymes (notably pancreatin) was partially limited when using the SNEDDS formulation. Overall, we successfully elaborated SNEDDS with a monodispersed size of approximately 62 nm, loaded with IP-DHA at 99% efficiency. These SNEDDS were showed to delay the degradation of the drug by the intestinal enzyme. The biological efficiency of free IP-DHA and SNEDDS-IP-DHA was then evaluated in vivo on ABCA4KO and BALB/c mice after oral administration and the induction of retinal degeneration.

### 3.4. In Vivo Comparison of the Effectiveness of Free IP-DHA vs. SNEDDS-IP-DHA on Mice after Oral Administration

In this in vivo study, we decided to fully evaluate the therapeutic efficacy of IP-DHA on STGD1 and dry AMD. Thus, we selected the appropriate mice models of these diseases and conducted investigations on the functionality and morphology of the photoreceptors after inducing retinal degeneration with acute and chronic via electroretinography and histological analysis.

#### 3.4.1. Acute Light-Induced Retinal Degeneration in ABCA4KO Mice

We used ABCA4KO mice, animal models of Stargardt’s disease [25], for this in vivo evaluation. The mice were kept in the dark for 24 h before the induction of retinal degeneration to regenerate the visual pigment (opsin + 11cRAL). We induced photoreceptor degeneration by exposing the mice to intense white light (20,000 Lux) for 90 min. Light-induced retinal photoreceptor degeneration on albino mice is a commonly used method of studying potential treatments on dry AMD [50,51]. The state of the retina was evaluated 5 days after exposure, with functional analysis via electroretinography (ERG), and a histological analysis. The mice were maintained in the dark during the 5 days. The effects of IP-DHA (100 mg/kg) and SNEDDS-IP-DHA (17, 50, and 100 mg/kg) were evaluated by force-feeding the mice 60 min before light exposure.

#### 3.4.2. Protection of Free IP-DHA and SNEDDS-IP-DHA against Acute Light-Induced Retinal Degeneration on ABCA4KO Mice Evaluated via Electroretinography

**Figure 7:**
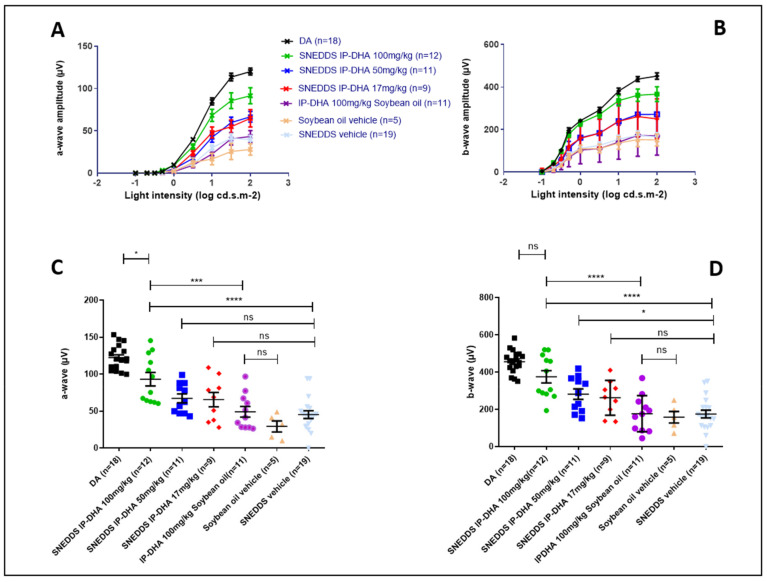
Light intensity of a- (**A**) and b- (**B**) wave and maximum light intensity of a- (**C**) and b- (**D**) wave of ABCA4KO mice after oral administration of various pretreatments and retinal degeneration induced through acute light stress. One-way ANOVA with Bonferroni’s multicomparison test: * *p* < 0.05, *** *p* < 0.001, **** *p* < 0.0001, ns: not significant.

ERG analysis is used to determine the electrical activity of the retina. The results showed the amplitude of a- and b-waves at different light intensities (Figure 7A,B). The maximum of the a- and b-amplitudes with the vehicles IP-DHA and SNEDDS-IP-DHA are represented in Figure 7C,D. Dark-adapted (DA) mice that were not exposed to light were considered to have 100% photoreceptor activity (positive control). Mice that were force-fed the vehicles (soybean oil and SNEDDS) were not protected from light-induced degeneration, and lost 76 and 63% of their activity, respectively (DA vs. soybean oil/SNEDDS, (Figure 7C). IP-DHA solubilized in soybean oil and administered at 100 mg/kg did not show significant protection compared to the vehicle (42 vs. 24% for a-wave and 39 vs. 35% for b-wave). SNEDDS-IP-DHA showed a dose-dependent pattern of protection: SNEDDS-IP-DHA at 17 mg/kg seemed to protect slightly better than the vehicles, but there was no significant difference. At 50 mg/kg, we observed a slight degree of protection compared to the SNEDDS vehicle for the a-wave (55% vs. 37% of photoreceptors remaining, no significant difference) and for the b-wave (62% vs. 38% of photoreceptors remaining, significant difference). At 100 mg/kg, this protection increased for a- and b-wave, respectively, to 76 and 82%. It is interesting to notice that by an IV route in similar experimental conditions, a dispersed formulation of IP-DHA associated with BSA that was injected at 30 mg/kg produced a similar degree of protection (80–85%) [8].

#### 3.4.3. Protection of Free IP-DHA and SNEDDS-IP-DHA against Acute Light-Induced Retinal Degeneration on ABCA4KO Mice Evaluated Using Histological Analysis

**Figure 8:**
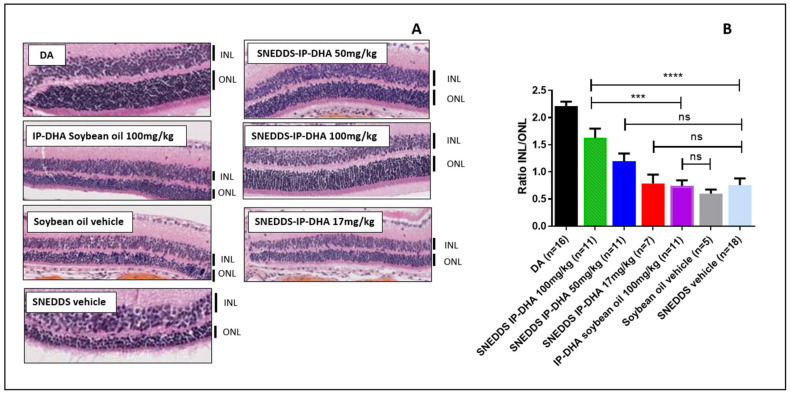
Images of histological analysis of ABCA4KO mice with various pretreatments after retinal degeneration induced by acute white light stress (**A**) and ratio of thickness of INL/ONL from this histological analysis (**B**). One-way ANOVA with Bonferroni’s multicomparison test: *** *p* < 0.001, **** *p* < 0.0001, ns: not significant.

To complete the ERG evaluation, we conducted a histological study. Indeed, the histological analysis is considered to be an anatomical analysis. Thus, we used it to determine the thickness of the inner (INL) and outer (ONL) nuclear layers of the retina. The ONL layer contains the photoreceptors, and degeneration and cell death are indicated by a decrease in the thickness of the ONL layer. The INL/ONL ratio was then measured with histological analysis. We have shown that SNEDDS-IP-DHA at 100 mg/kg effectively protected the activity of photoreceptors in ABCA4KO mice. With this histological analysis, we wanted to evaluate the structure of the ONL and INL layers. The results were similar to those observed with the ERG analysis (Figure 8). DA mice showed an INL/ONL ratio of 2.2: the ONL layer was approximately two-fold thicker than the INL layer in untreated mice. Vehicles (soybean oil and SNEDDS) and IP-DHA solubilized in soybean oil at 100 mg/kg did not show any protective effects, and presented with INL/ONL ratios of 0.6; 0.7, and 0.7, respectively. We found a similar dose-response effect with SNEDDS-IP-DHA-treated mice with ERG analysis: a slight improvement in the preservation of the thickness of the ONL layer in mice treated with 17 and 50 mg/kg, but no significant difference being observed with the vehicles. The protective effect was observed at a dose of 100 mg/kg with SNEDDS-IP-DHA (INL/ONL ratio: 1.6); i.e., a conservation of layer thickness of 73%. This protection was slightly lower than those obtained via an IV route at a lower dose of 30 mg/kg [8].

The correlation observed between the histological results and the ERG analyses revealed that IP-DHA had a protective effect on the integrity of the photoreceptors after the induction of acute light stress with white light in a mouse model of STDG1. This protective effect was observed after oral administration with a SNEDDS formulation. At the same dose (100 mg/kg), we observed a major difference in the protective effect with and without the formulation (SNEDDS-IP-DHA 100 mg/kg vs. IP-DHA 100 mg/kg in soybean oil), which proved that the formulation allowed for a significant increase in the bioavailability of the molecule, and thus, an increase in the amount of active molecule reaching the photoreceptors. SNEDDS vehicles did not show any protective effects, and therefore, it was not involved in the protective effect.

Previous studies have demonstrated the efficacy of various compounds in vivo via an oral route in an acute light-induced retinal degeneration model. Astaxantin that was dissolved in olive oil and orally administrated at 100 mg/kg to ddY mice partially preserve photoreceptors before the induction of retinal degeneration with white light (3 h at 8000 Lux) [52]. In another work, NSP-116 suspended in water with 0.5% sodium carboxymethyl cellulose (CMC) was administered orally at 30 mg/kg to ddY mice 30 min before inducing retinal degeneration with white light (8000 Lux for 3 h). NSP-116 was effective in protecting photoreceptors in mice. However the PK study displayed a rather low amount of NSP-166 in plasma (C_max_= 7 µg/mL for an administered dose of 25 mg/kg) [53]. In our study, as we demonstrated the importance of SNEDDS formulation to enhance the activity of IP-DHA (Figure 6 and Figure 7), an adapted formulation of Astaxantin and NSP-116 might increase their bioavailability and thus their activity.

#### 3.4.4. Chronic Light-Induced Retinal Degeneration on BALB/c Mice

The degeneration induced by white light allows us to highlight the protective effect of IP-DHA after a single gavage, and over a short period of time (90 min). To go further and to reach the real conditions of dry AMD (a long period of time and a low light intensity), we induced the degeneration of the photoreceptors of mice at low light intensity with blue LED light (1000 Lux) over a longer period of time (7 days) with an exposure/non-exposure cycle of 12/12 h, and without pupil dilatation. The protective effect of SNEDDS-IP-DHA was evaluated at 100 mg/kg by varying the number of daily doses (details in Section 2.4.4). We used BALB/c mice instead of ABCA4KO mice. ABCA4KO mice are models of Stargardt’s disease, and the BALB/c mice represent an AMD model that is not linked to the Abca4 gene deletion, which corresponds better with the use of chronic retinal degeneration. After the preliminary tests using mice that had been exposed over 7 days at 12 h/day to white or blue LED light at ≈1000 Lux, we observed that white light was not enough to induce a sufficient degree of degeneration (15%) compared to blue LED light at the same intensity (74%) (data not shown), so blue LED light was chosen instead of white light to induce chronic degeneration. Several studies have already used blue light to induce photoreceptor degeneration as a model for AMD studies [54], and the impact of blue light in AMD has also been demonstrated [55,56].

#### 3.4.5. Protection of Free IP-DHA and SNEDDS-IP-DHA against Chronic Light-Induced Retinal Degeneration on BALB/c Mice Evaluated Using Electroretinography

**Figure 9:**
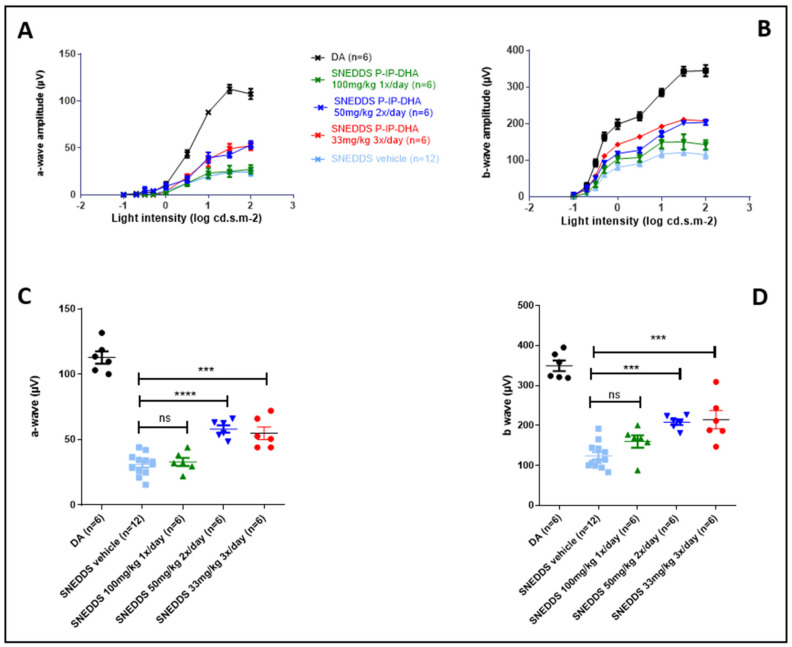
Light intensity of a- (**A**) and b- (**B**) wave of BALB/c mice, and maximum light intensity of a- (**C**) and b- (**D**) wave with oral administration of various pretreatments after retinal degeneration-induced chronic blue light stress. One-way ANOVA with Bonferroni’s multicomparison test: *** *p* < 0.001, **** *p* < 0.0001, ns: not significant.

ERG results demonstrated that SNEDDS vehicles did not protect from blue light (≈1000 Lux), with 27% of photoreceptors remaining with SNEDDS compared to DA (100%). At a single daily dose of 100 mg/kg, SNEDDS-IP-DHA did not show an increase in any protective effect (29% of photoreceptors remaining). When maintaining the same daily dose but giving lower doses at shorter time intervals (2 × 50 mg/kg/day and 3 × 33 mg/kg/day), we observed a significant increase in the protection of photoreceptor activity. After the induction of degeneration with chronic light stress under low blue light intensities, 52% and 49% of photoreceptors remained, respectively (Figure 9).

#### 3.4.6. Protection of Free IP-DHA and SNEDDS-IP-DHA against Chronic Light-Induced Retinal Degeneration on BALB/c Mice Evaluated via Histological Analysis

The results of ERG were completed with the histological analysis. The latter highlighted a correlation with the results obtained from ERG in a similar manner to the acute model. The histological analysis confirmed that the number of photoreceptors observed decreased in the ONL layer of mice treated with vehicle SNEDDS and SNEDDS-IP-DHA at 1 × 100 mg/kg/day and subjected to chronic light stress under blue light. The analysis also confirmed that the protection of photoreceptor integrity was observed with mice with treatments of 2 × 50 mg/kg/day and 3 × 33 mg/kg/day. The percentages of photoreceptors remaining with this analysis were 69% and 51%, respectively.

Analyses with this model of chronic degeneration allowed us to observe that the protection of IP-DHA was not only dose-dependent, but also frequency-dependent. The fact that no protection was observed with a once-a-day administration at 100 mg/kg suggested that IP-DHA had a reduced half-time; it was degraded too quickly in the blood and/or eye, and it did not effectively protect the eye for the duration of the exposure (12 h/day). On the other hand, with an administration frequency of two or three times a day, a more important protection was noticed, which suggested that the quantity of IP-DHA in the eye during the day remained sufficient for protection over the whole duration of the exposure. In view of these results, it is probable that a more frequent administration at higher doses or an extended-release form could further improve the level of protection.

**Figure 10:**
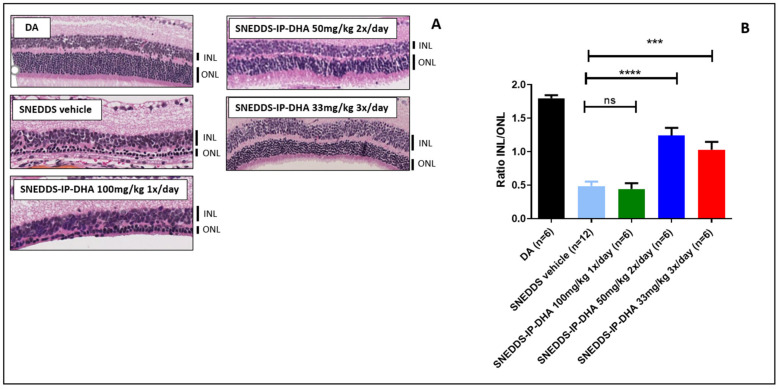
Ratio of thickness of INL/ONL of BALB/c mice with various pretreatments from histological analysis after retinal degeneration induced by chronic blue light stress (**A**,**B**). One-way ANOVA with Bonferroni’s multicomparison test: *** *p* < 0.001, **** *p* < 0.0001, ns: not significant.

Previous studies have evaluated the therapeutic effect in vivo of different molecules by inducing retinal degeneration with chronic light stress under blue light. For example, lutein orally administered at 25 mg/kg/day with corn oil demonstrated its efficacy for protecting photoreceptors for 30 days, with 1.5 h/day (1500 Lux) of light exposure after pretreatment on rats [57]. In another study, the authors administered Cistanche Tubulosa orally to rats at a rate of 100 mg/kg/day for 74 days, induced degeneration with blue light (150 Lux) 3 h/day during the last 60 days, and demonstrated the protective effect of Cistanche Tubulosa in vivo [58]. These studies showed that orally administrated antioxidant compounds can have a beneficial effect in the treatment of AMD; however, it would be interesting to study the effects of these compounds over longer exposure times to better imitate the pathophysiological conditions of AMD. For comparison, our study demonstrated the ability of IP-DHA to partially protect the retina 12 h in a row (Figure 9 and Figure 10). Further studies will be conducted to optimize the doses administrated and the frequency of administration per day. After determining the ideal pharmaceutical form and dosage, it would also be interesting to conduct a study over a longer period of time.

## 4. Conclusions

In this study, we aimed at evaluating the potential of a new lipophenol drug, IP-DHA, to prevent dry AMD and STDG1. For this, we first fully analyzed the IP-DHA physico-chemical properties in order to select the best-suited formulation for its efficient oral delivery. IP-DHA was found to be poorly soluble and poorly permeable, belonging to the BCS class IV drugs, and also highly metabolized by the liver. Thus, our choice of formulation was oriented towards lipid-based SNEDDS formulations composed of Phosal^®^ 53 MCT Labrasol^®^, and Transcutol HP^®^. After a pseudo-ternary phase diagram investigation, the ratio was optimized to 25/60/15 oil phase/surfactant/co-surfactant, leading to the formation of small and homogeneous NE of approximately 52 nm mean diameter. An IP-DHA loading of 30 mg/mL was successfully performed with a low impact on droplet size, and the homogeneity of NE and loaded SNEDDS were proven to be robust to dilution and to reduce the impact of intestinal enzymes on the degradation of the drug. The full in vivo assessment of IP-DHA SNEDDS biological activity demonstrated that the formulation allowed for the sufficient absorption of IP-DHA to protect the mice from both acute and chronically induced degeneration. Overall, the developed SNEDDS-IP-DHA formulation holds promising future preventive treatments via the oral route of the targeted diseases. In addition, other routes of administration such as topical instillation are currently being investigated.

## Figures and Tables

**Table 1:** Composition of SGF and ISF used for the stability study.

SGF	ISF
2 g NaCl	5 mL pancreatin (4.80 mg/mL)
7 mL HCl 37%	8 mL bile salts (5 mg/mL)
3.2 g pepsin	2 mL CaCl_2_ (83.5 mg/mL)
1 L water	PBS pH = 7.4
pH = 1.5	pH = 7.4

**Table 2:** Solubility, permeability, and stability of IP-DHA on microsomes (*n* = 3).

Characteristics Analyzed	Results
Molecular weight	478 g/mol
Log P	7.91
Solubility in PBS, pH 7.4	11.54 ± 1.53 µg/mL
Solubility in SGF	7.39 ± 0.26 µg/mL
Solubility in ISF	10.19 ± 0.12 µg/mL
A–B permeability (Caco-2, pH 6.5/7.4)	1.0 ± 0.14 10^−6^ cm/s
B–A permeability (Caco-2, pH 6.5/7.4)	0.7 ± 0.05 10^−6^ cm/s
Stability on human microsomes	T_1/2_: 10 min

**Table 3:** Influence of excipient ratio on mean size of NE (SNEDDS diluted in water at 1:1000, *n* = 3).

Ratio (% *w*/*w*/*w*) Phosal^®^ 53 MCT, Labrasol^®^, Transcutol HP^®^	Mean Size of NE Droplets (nm)	PDI
5/50/45	78.43 ± 9.86	0.20 ± 0.08
5/85/10	79.31 ± 2.8	0.14 ± 0.12
10/85/5	96.75 ± 16.00	0.21 ± 0.09
20/65/15	57.60 ± 4.14	0.10 ± 0.03
20/60/20	69.18 ± 4.44	0.25 ± 0.08
25/60/15	53.53 ± 4.5	0.15 ± 0.03
30/60/10	57.15 ± 7.07	0.12 ± 0.02
35/60/15	NA	NA

**Table 4:** Size, PDI, and zeta potential of NE or NE-IP-DHA in water after dilution of SNEDDS and SNEDDS-IP-DHA at 30 mg/mL (*n* = 3).

	NE	NE-IP-DHA
Droplet Size (diluted 1:1000)	53.53 ± 4.50 nm	62.40 ± 3.37 nm
PDI	0.15 ± 0.03	0.11 ± 0.02
Zeta potential (diluted 1:100)	−8.04 ± 1.73 mV	−22.03 ± 3.29 mV

## Data Availability

Data supporting reported results can be found at ICGM, Univ Montpellier, CNRS, ENSCM, 34000 Montpellier, France and at Institut des Neurosciences de Montpellier, INSERM U1051, 34000 Montpellier, France.

## Supplementary Materials

The following supporting information can be downloaded at: https://www.mdpi.com/article/10.3390/pharmaceutics14051029/s1, Figure S1: XRD analysis of IP-DHA, Figure S2: DSC TGA analysis of IP-DHA, Figure S3: Stability on human microsomes of IP-DHA.

## References

[1] Deng Y., Qiao L., Du M., Qu C., Wan L., Li J., Huang L. (2021). Age-Related Macular Degeneration: Epidemiology, Genetics, Pathophysiology, Diagnosis, and Targeted Therapy. Genes Dis..

[2] Wong W.L., Su X., Li X., Cheung C.M.G., Klein R., Cheng C.-Y., Wong T.Y. (2014). Global Prevalence of Age-Related Macular Degeneration and Disease Burden Projection for 2020 and 2040: A Systematic Review and Meta-Analysis. Lancet Glob. Health.

[3] Zhou J., Jang Y.P., Kim S.R., Sparrow J.R. (2006). Complement Activation by Photooxidation Products of A2E, a Lipofuscin Constituent of the Retinal Pigment Epithelium. Proc. Natl. Acad. Sci. USA.

[4] Maeda T., Golczak M., Maeda A. (2012). Retinal Photodamage Mediated by All-*Trans*-Retinal ^†^. Photochem. Photobiol..

[5] Wang P., Chin E.K., Almeida D. (2021). Antioxidants for the Treatment of Retinal Disease: Summary of Recent Evidence. Clin. Ophthalmol..

[6] Crauste C., Vigor C., Brabet P., Picq M., Lagarde M., Hamel C., Durand T., Vercauteren J. (2014). Synthesis and Evaluation of Polyunsaturated Fatty Acid-Phenol Conjugates as Anti-Carbonyl-Stress Lipophenols: Polyunsaturated Fatty Acid-Phenol Conjugates. Eur. J. Org. Chem..

[7] Cia D., Cubizolle A., Crauste C., Jacquemot N., Guillou L., Vigor C., Angebault C., Hamel C.P., Vercauteren J., Brabet P. (2016). Phloroglucinol Protects Retinal Pigment Epithelium and Photoreceptor against All- *Trans*-Retinal-Induced Toxicity and Inhibits A2E Formation. J. Cell. Mol. Med..

[8] Taveau N., Cubizolle A., Guillou L., Pinquier N., Moine E., Cia D., Kalatzis V., Vercauteren J., Durand T., Crauste C. (2020). Preclinical Pharmacology of a Lipophenol in a Mouse Model of Light-Induced Retinopathy. Exp. Mol. Med..

[9] Kheawfu K., Pikulkaew S., Rades T., Müllertz A., Okonogi S. (2018). Development and Characterization of Clove Oil Nanoemulsions and Self-Microemulsifying Drug Delivery Systems. J. Drug Deliv. Sci. Technol..

[10] Okonogi S., Phumat P., Khongkhunthian S., Chaijareenont P., Rades T., Müllertz A. (2021). Development of Self-Nanoemulsifying Drug Delivery Systems Containing 4-Allylpyrocatechol for Treatment of Oral Infections Caused by *Candida Albicans*. Pharmaceutics.

[11] Savla R., Browne J., Plassat V., Wasan K.M., Wasan E.K. (2017). Review and Analysis of FDA Approved Drugs Using Lipid-Based Formulations. Drug Dev. Ind. Pharm..

[12] Udaya Sakthi M., Lobo F.J.R., Uppuluri K.B. (2013). Self Nano Emulsifying Drug Delivery Systems for Oral Delivery of Hydrophobic Drugs. Biomed. Pharmacol. J..

[13] Date A.A., Desai N., Dixit R., Nagarsenker M. (2010). Self-Nanoemulsifying Drug Delivery Systems: Formulation Insights, Applications and Advances. Nanomedicine.

[14] Kuentz M. (2012). Lipid-Based Formulations for Oral Delivery of Lipophilic Drugs. Drug Discov. Today Technol..

[15] Kalepu S., Manthina M., Padavala V. (2013). Oral Lipid-Based Drug Delivery Systems—An Overview. Acta Pharm. Sin. B.

[16] Patel V., Lalani R., Bardoliwala D., Ghosh S., Misra A. (2018). Lipid-Based Oral Formulation Strategies for Lipophilic Drugs. AAPS PharmSciTech.

[17] Neslihan Gursoy R., Benita S. (2004). Self-Emulsifying Drug Delivery Systems (SEDDS) for Improved Oral Delivery of Lipophilic Drugs. Biomed. Pharmacother..

[18] Cherniakov I., Domb A.J., Hoffman A. (2015). Self-Nano-Emulsifying Drug Delivery Systems: An Update of the Biopharmaceutical Aspects. Expert Opin. Drug Deliv..

[19] Yoo J.H., Shanmugam S., Thapa P., Lee E.-S., Balakrishnan P., Baskaran R., Yoon S.-K., Choi H.-G., Yong C.S., Yoo B.K. (2010). Novel Self-Nanoemulsifying Drug Delivery System for Enhanced Solubility and Dissolution of Lutein. Arch. Pharm. Res..

[20] Shanmugam S., Baskaran R., Balakrishnan P., Thapa P., Yong C.S., Yoo B.K. (2011). Solid Self-Nanoemulsifying Drug Delivery System (S-SNEDDS) Containing Phosphatidylcholine for Enhanced Bioavailability of Highly Lipophilic Bioactive Carotenoid Lutein. Eur. J. Pharm. Biopharm..

[21] Sokkula S.R., Gande S. (2020). A Comprehensive Review on Self-Nano Emulsifying Drug Delivery Systems: Advancements & Applications. Int. J. Pharm. Sci. Drug Res..

[22] Nayak K., Misra M. (2018). A Review on Recent Drug Delivery Systems for Posterior Segment of Eye. Biomed. Pharmacother..

[23] Chekroud K., Guillou L., Grégoire S., Ducharme G., Brun E., Cazevieille C., Bretillon L., Hamel C.P., Brabet P., Pequignot M.O. (2012). Fatp1 Deficiency Affects Retinal Light Response and Dark Adaptation, and Induces Age-Related Alterations. PLoS ONE.

[24] Chen Y., Okano K., Maeda T., Chauhan V., Golczak M., Maeda A., Palczewski K. (2012). Mechanism of All-Trans-Retinal Toxicity with Implications for Stargardt Disease and Age-Related Macular Degeneration. J. Biol. Chem..

[25] Lenis T.L., Hu J., Ng S.Y., Jiang Z., Sarfare S., Lloyd M.B., Esposito N.J., Samuel W., Jaworski C., Bok D. (2018). Expression of ABCA4 in the Retinal Pigment Epithelium and Its Implications for Stargardt Macular Degeneration. Proc. Natl. Acad. Sci. USA.

[26] Yang E.-J., Ahn S., Ryu J., Choi M.-S., Choi S., Chong Y.H., Hyun J.-W., Chang M.-J., Kim H.-S. (2015). Phloroglucinol Attenuates the Cognitive Deficits of the 5XFAD Mouse Model of Alzheimer’s Disease. PLoS ONE.

[27] Shindou H., Koso H., Sasaki J., Nakanishi H., Sagara H., Nakagawa K.M., Takahashi Y., Hishikawa D., Iizuka-Hishikawa Y., Tokumasu F. (2017). Docosahexaenoic Acid Preserves Visual Function by Maintaining Correct Disc Morphology in Retinal Photoreceptor Cells. J. Biol. Chem..

[28] Cubizolle A., Cia D., Moine E., Jacquemot N., Guillou L., Rosell M., Angebault-Prouteau C., Lenaers G., Meunier I., Vercauteren J. (2020). Isopropyl-phloroglucinol-DHA Protects Outer Retinal Cells against Lethal Dose of All- *Trans*-retinal. J. Cell. Mol. Med..

[29] Youhanna S., Lauschke V.M. (2021). The Past, Present and Future of Intestinal In Vitro Cell Systems for Drug Absorption Studies. J. Pharm. Sci..

[30] Amidon G.L., Lennernäs H., Shah V.P., Crison J.R. (1995). A Theoretical Basis for a Biopharmaceutic Drug Classification: The Correlation of in Vitro Drug Product Dissolution and in Vivo Bioavailability. Pharm. Res..

[31] Shrestha H., Bala R., Arora S. (2014). Lipid-Based Drug Delivery Systems. J. Pharm..

[32] Gupta S., Kesarla R., Omri A. (2013). Formulation Strategies to Improve the Bioavailability of Poorly Absorbed Drugs with Special Emphasis on Self-Emulsifying Systems. ISRN Pharm..

[33] Krstić M., Medarević Đ., Đuriš J., Ibrić S. (2018). Self-Nanoemulsifying Drug Delivery Systems (SNEDDS) and Self-Microemulsifying Drug Delivery Systems (SMEDDS) as Lipid Nanocarriers for Improving Dissolution Rate and Bioavailability of Poorly Soluble Drugs. Lipid Nanocarriers for Drug Targeting.

[34] Buya A.B., Beloqui A., Memvanga P.B., Préat V. (2020). Self-Nano-Emulsifying Drug-Delivery Systems: From the Development to the Current Applications and Challenges in Oral Drug Delivery. Pharmaceutics.

[35] Basalious E.B., Shawky N., Badr-Eldin S.M. (2010). SNEDDS Containing Bioenhancers for Improvement of Dissolution and Oral Absorption of Lacidipine. I: Development and Optimization. Int. J. Pharm..

[36] Morakul B. (2020). Self-Nanoemulsifying Drug Delivery Systems (SNEDDS): An Advancement Technology for Oral Drug Delivery. Pharm. Sci. Asia.

[37] Chatterjee B., Hamed Almurisi S., Ahmed Mahdi Dukhan A., Mandal U.K., Sengupta P. (2016). Controversies with Self-Emulsifying Drug Delivery System from Pharmacokinetic Point of View. Drug Deliv..

[38] O’Driscoll C.M. (2002). Lipid-Based Formulations for Intestinal Lymphatic Delivery. Eur. J. Pharm. Sci..

[39] Charman W.N.A., Stella V.J. (1986). Estimating the Maximal Potential for Intestinal Lymphatic Transport of Lipophilic Drug Molecules. Int. J. Pharm..

[40] Sha X., Yan G., Wu Y., Li J., Fang X. (2005). Effect of Self-Microemulsifying Drug Delivery Systems Containing Labrasol on Tight Junctions in Caco-2 Cells. Eur. J. Pharm. Sci..

[41] McCartney F., Jannin V., Chevrier S., Boulghobra H., Hristov D.R., Ritter N., Miolane C., Chavant Y., Demarne F., Brayden D.J. (2019). Labrasol^®^ Is an Efficacious Intestinal Permeation Enhancer across Rat Intestine: Ex Vivo and in Vivo Rat Studies. J. Control. Release.

[42] Shen H., Zhong M. (2006). Preparation and Evaluation of Self-Microemulsifying Drug Delivery Systems (SMEDDS) Containing Atorvastatin. J. Pharm. Pharmacol..

[43] Patel M.H., Sawant K.K. (2019). Self Microemulsifying Drug Delivery System of Lurasidone Hydrochloride for Enhanced Oral Bioavailability by Lymphatic Targeting: In Vitro, Caco-2 Cell Line and in Vivo Evaluation. Eur. J. Pharm. Sci..

[44] Constantinides P.P., Scalart J.-P., Lancaster C., Marcello J., Marks G., Ellens H., Smith P.L. (1994). Formulation and Intestinal Absorption Enhancement Evaluation of Water-in-Oil Microemulsions Incorporating Medium-Chain Glycerides. Pharm. Res..

[45] Rao S.V.R., Yajurvedi K., Shao J. (2008). Self-Nanoemulsifying Drug Delivery System (SNEDDS) for Oral Delivery of Protein Drugs. Int. J. Pharm..

[46] Muller R.H., Jacobs C., Kayser O. (2001). Nanosuspensions as Particulate Drug Formulations in Therapy Rationale for Development and What We Can Expect for the Future. Adv. Drug Deliv. Rev..

[47] AboulFotouh K., Allam A.A., El-Badry M., El-Sayed A.M. (2017). Development and in Vitro/in Vivo Performance of Self-Nanoemulsifying Drug Delivery Systems Loaded with Candesartan Cilexetil. Eur. J. Pharm. Sci..

[48] Yu A., Jackson T., Tsume Y., Koenigsknecht M., Wysocki J., Marciani L., Amidon G.L., Frances A., Baker J.R., Hasler W. (2017). Mechanistic Fluid Transport Model to Estimate Gastrointestinal Fluid Volume and Its Dynamic Change Over Time. AAPS J..

[49] Torcello-Gómez A., Maldonado-Valderrama J., de Vicente J., Cabrerizo-Vílchez M.A., Gálvez-Ruiz M.J., Martín-Rodríguez A. (2011). Investigating the Effect of Surfactants on Lipase Interfacial Behaviour in the Presence of Bile Salts. Food Hydrocoll..

[50] Izawa H., Inoue Y., Ohno Y., Ojino K., Tsuruma K., Shimazawa M., Hara H. (2015). Protective Effects of Antiplacental Growth Factor Antibody Against Light-Induced Retinal Damage in Mice. Investig. Opthalmol. Vis. Sci..

[51] Ortega J.T., Parmar T., Golczak M., Jastrzebska B. (2021). Protective Effects of Flavonoids in Acute Models of Light-Induced Retinal Degeneration. Mol. Pharmacol..

[52] Otsuka T., Shimazawa M., Nakanishi T., Ohno Y., Inoue Y., Tsuruma K., Ishibashi T., Hara H. (2013). The Protective Effects of a Dietary Carotenoid, Astaxanthin, Against Light-Induced Retinal Damage. J. Pharmacol. Sci..

[53] Izawa H., Shimazawa M., Inoue Y., Uchida S., Moroe H., Tsuruma K., Hara H. (2016). Protective Effects of NSP-116, a Novel Imidazolyl Aniline Derivative, against Light-Induced Retinal Damage in Vitro and in Vivo. Free Radic. Biol. Med..

[54] Wielgus A.R., Collier R.J., Martin E., Lih F.B., Tomer K.B., Chignell C.F., Roberts J.E. (2010). Blue Light Induced A2E Oxidation in Rat Eyes—Experimental Animal Model of Dry AMD. Photochem. Photobiol. Sci..

[55] King A., Gottlieb E., Brooks D.G., Murphy M.P., Dunaief J.L. (2007). Mitochondria-Derived Reactive Oxygen Species Mediate Blue Light-Induced Death of Retinal Pigment Epithelial Cells. Photochem. Photobiol..

[56] Algvere P.V., Marshall J., Seregard S. (2006). Age-Related Maculopathy and the Impact of Blue Light Hazard. Acta Ophthalmol. Scand..

[57] Yang J., Li D., Zhang Y., Zhang L., Liao Z., Aihemaitijiang S., Hou Y., Zhan Z., Xie K., Zhang Z. (2020). Lutein Protected the Retina from Light Induced Retinal Damage by Inhibiting Increasing Oxidative Stress and Inflammation. J. Funct. Foods.

[58] Wu M.-R., Lin C.-H., Ho J.-D., Hsiao G., Cheng Y.-W. (2018). Novel Protective Effects of Cistanche Tubulosa Extract Against Low-Luminance Blue Light-Induced Degenerative Retinopathy. Cell. Physiol. Biochem..

