# Supplementary material for: Formulation and Evaluation of SNEDDS Loaded with Original Lipophenol for the Oral Route to Prevent Dry AMD and Stragardt’s Disease
Source: Pharmaceutics. 2022 May 10;14(5):1029. doi: 10.3390/pharmaceutics14051029 (PMC9147958; doi:10.3390/pharmaceutics14051029)
Supplement: Supplementary file 1 [file pharmaceutics-14-01029-s001.zip › pharmaceutics-1707269-supplementary.pdf]

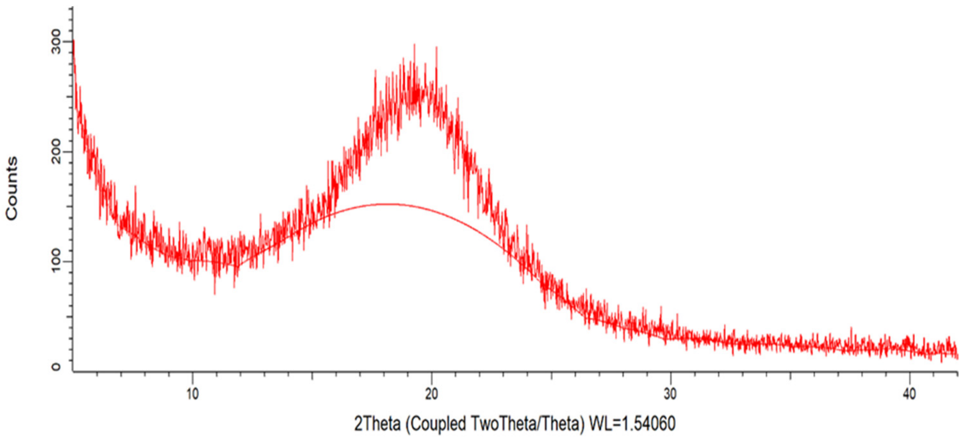

Figure S1. XRD analysis of IP-DHA.

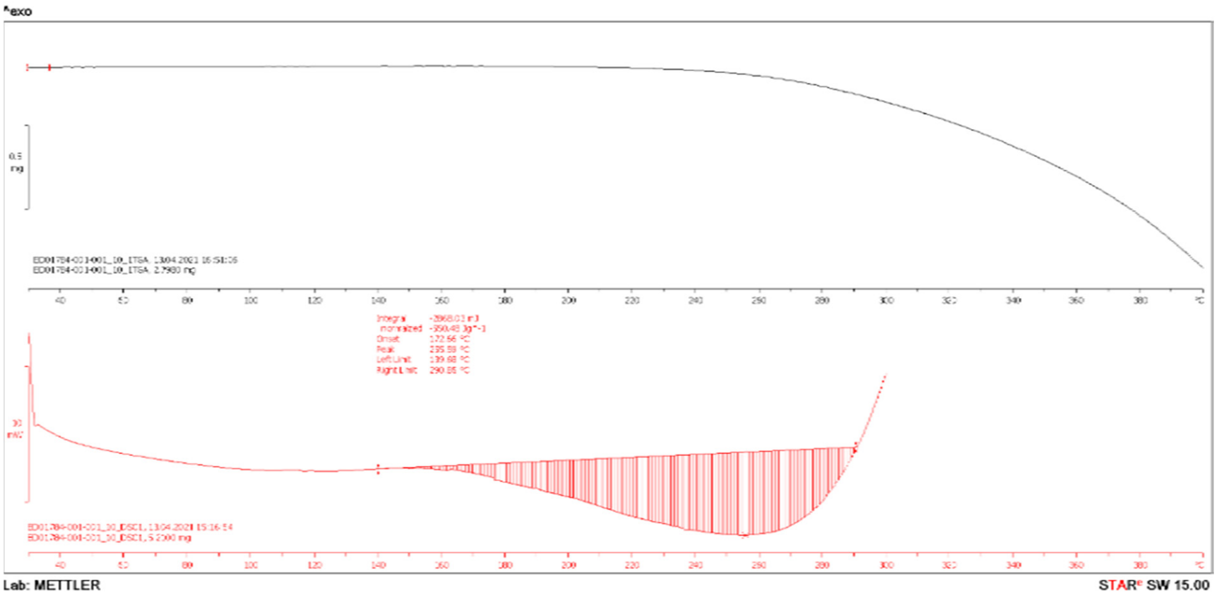

Figure S2. DSC TGA analysis of IP-DHA.

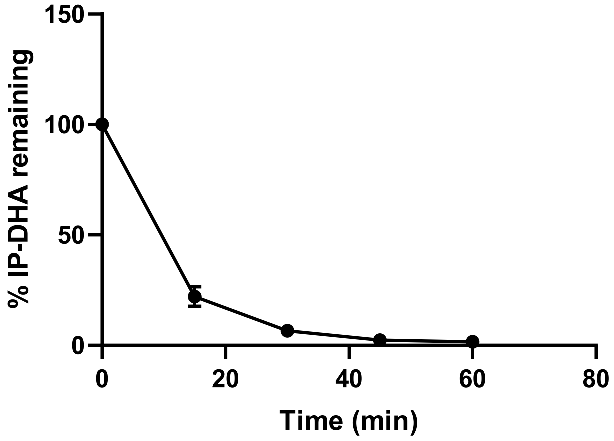

Figure S3. Stability on human microsomes of IP-DHA.
